# Supplementary material for: MetaRibo-Seq measures translation in microbiomes
Source: Nat Commun. 2020 Jun 29;11:3268. doi: 10.1038/s41467-020-17081-z (PMC7324362; doi:10.1038/s41467-020-17081-z)
Supplement: Supplementary file 10 — Supplementary Data 7 [file 41467_2020_17081_MOESM10_ESM.zip › File2/Confidence_High_Taxonomy/346203_out.krona.html]

Javascript must be enabled to view this page.

members
magnitude
magnitudeUnassigned
count
unassigned
taxon
rank

346203\_out

18

2
18
superkingdom

phylum
18
1239

class
18
186801

order
18
186802

186803
18
family

18
genus
841


SRS013158\_contig\_number\_contig-100\_329.42835SRS013940\_contig\_number\_7252SRS014948\_contig\_number\_14859SRS043001\_contig\_number\_contig-100\_75.68752SRS045739\_contig\_number\_contig-100\_526.55630SRS049900\_contig\_number\_17718SRS062427\_contig\_number\_contig-100\_401.401SRS063040\_contig\_number\_44931SRS065504\_contig\_number\_contig-100\_2322.222083SRS075341\_contig\_number\_contig-100\_1580.155360SRS077641\_contig\_number\_contig-100\_2150.117805SRS104975\_contig\_number\_contig-100\_2724.109921SRS1055049\_contig\_number\_5142SRS1055067\_contig\_number\_9383SRS147919\_contig\_number\_20779SRS148091\_contig\_number\_8710SRS148721\_contig\_number\_23925SRS149879\_contig\_number\_22500
166486
18
species
